# Fall prevention in community-dwelling adults with mild to moderate cognitive impairment: a systematic review and meta-analysis

**DOI:** 10.1186/s12877-021-02641-9

**Published:** 2021-12-10

**Authors:** M. Racey, M. Markle-Reid, D. Fitzpatrick-Lewis, M. U. Ali, H. Gagne, S. Hunter, J. Ploeg, R. Sztramko, L. Harrison, R. Lewis, M. Jovkovic, D. Sherifali

**Affiliations:** 1 McMaster Evidence Review and Synthesis Team; and School of Nursing, Faculty of Health Sciences, McMaster University, Hamilton, Canada; 2 School of Nursing, Faculty of Health Sciences, McMaster University; and Scientific Director, Aging, Community and Health Research Unit, McMaster University, Hamilton, Canada; 3 McMaster Evidence Review and Synthesis Team and Department of Clinical Epidemiology & Biostatistics, Faculty of Health Sciences, McMaster University, Hamilton, Canada; 4 Injury Prevention, Ontario Neurotrauma Foundation, Toronto, Canada; 5 School of Physical Therapy, University of Western Ontario, London, Ontario Canada; 6 School of Nursing, McMaster University and Aging, Community and Health Research Unit, McMaster University, Hamilton, Canada; 7 Geriatric Medicine, McMaster University, Hamilton, Canada; 8 Caregiver Partner, Hamilton, Canada; 9 McMaster Evidence Review and Synthesis Team, Hamilton, Canada

**Keywords:** Fall prevention, Cognitive impairment, Older adults, Systematic review, Meta analysis

## Abstract

**Background:**

Cognitive impairment (CI) increases an individual’s risk of falls due to the role cognition plays in gait control. Older adults with dementia fall 2–3 times more than cognitively healthy older adults and 60–80% of people with dementia fall annually. Practitioners require evidence-based fall prevention best practices to reduce the risk of falls in cognitively impaired adults living in the community.

**Methods:**

We conducted a systematic review and meta-analysis to identify the effectiveness of primary and secondary fall prevention interventions in reducing falls and fear of falling, and improving gait, balance, and functional mobility. We searched 7 databases for fall prevention interventions involving community-dwelling adults ≥50 years with mild to moderate CI. Reviewers screened citations, extracted data, and assessed risk of bias and certainty of evidence (GRADE). We assessed statistical and methodological heterogeneity and performed a meta-analysis of studies including subgroup analysis based on intervention and risk of bias groupings.

**Results:**

Five hundred nine community-dwelling adults (mean age 67.5 to 84.0 years) with mild to moderate CI from 12 randomized or clinical controlled trials (RCTs/CCTs) were included in this review. Eight studies were exercise interventions, 3 were multifactorial, and 1 provided medication treatment. Fall prevention interventions had significant effects of medium magnitude on fear of falling (standardized mean difference (SMD) -0.73 [− 1.10, − 0.36]), balance (SMD 0.66 [0.19, 1.12]), and functional mobility measured as Timed Up and Go test (SMD -0.56 [− 0.94, − 0.17]) and significant effects of small magnitude on gait control (SMD 0.26 [0.08, 0.43]) all with moderate certainty of evidence. The meta-analysis showed no significant effects for falls (number of events or falls incidence). Sub-analysis showed that exercise and low risk of bias studies remained significant for balance and perceived risk of falls.

**Conclusion:**

The effect of fall prevention interventions on direct outcomes, such as falls, remains unclear in cognitively impaired individuals. Exercise interventions are effective at improving fall risk factors, however, high quality studies with longer follow-up and adequate sample sizes are needed to determine their effectiveness on falls directly. There remains a gap in terms of effective fall prevention interventions for older adults with CI.

**Supplementary Information:**

The online version contains supplementary material available at 10.1186/s12877-021-02641-9.

## Background

Falls affect more than 30% of the adult population aged 65 years and older [1] and can result in negative health outcomes and severe injuries such as traumatic brain injuries and even death [2]. Falls in older adults are multifactorial with over 400 identifiable risk factors such as physical deficits, reduced lower-limb strength, gait and balance impairment, previous slips or trips, difficulty in activities of daily living (ADLs), functional impairment, prescribed drugs and medications, environmental hazards, and others [3–5]. Not only can falls result in injuries, but they can also lead to negative mental health outcomes such as fear of falling, loss of autonomy, poor quality of life, greater isolation, confusion, and depression. Falls also cost our public health system as they are the leading cause of injury-related admissions to acute care hospitals and in-hospital deaths. With an aging population, the cost of fall injuries to seniors in Canada is estimated to rise from $2.4 billion a year in direct healthcare costs [2] to $240 billion by 2040 [4].

Cognitive impairment occurs on a continuum from mild, moderate, to severe states. Mild cognitive impairment (MCI) is an intermediate clinical state between normal cognitive aging and dementia, and it precedes and leads to dementia in many cases [6, 7]. It is defined by subjective cognitive complaints and measurable cognitive decline in the absence of interference with daily function that is not due to age [8]. The term dementia is used when individuals have subjective cognitive complaints, measurable cognitive decline, and functional impairment. The terms Mild or Moderate Dementia are used when employing functional or cognitive assessments to distinguish where along the spectrum from mild to severe cognitive impairment (or dementia) an individual may exist. Cognitive impairment is often due to neurodegenerative diseases that are associated with advancing age, but it can also occur due to head trauma, stroke, or other diseases and affect those at any age [7].

Cognitive impairment (CI) is an important risk factor for experiencing falls. Any adult with cognitive impairment can also experience falls at an increased rate compared to the general population that is cognitively healthy [5]. In fact, older adults with dementia fall two to three times more than cognitively healthy older adults [9] and 60–80% of people with dementia fall annually [3]. This is due to the role cognition plays in the control of gait and the altered gait patterns that adults with cognitive impairment demonstrate [3] including reduced speed and increased stride variability. Research shows that the regions of the brain involved in cognitive functioning and memory are required to coordinate mobility, balance, and gait. Cognition could influence gait through judgement, reaction time, and/or psychomotor control but which of these or in what combination remains unclear [10, 11].

Fall prevention strategies have demonstrated success in reducing risk factors and rate of falls in the cognitively healthy older adults [12]. Multifactorial interventions, which most commonly includes exercise, as well as exercise as a standalone intervention, have been shown to reduce the rate of falls and risk of falls in community-dwelling older adults [12, 13]. However, these strategies have not translated successfully to adults with cognitive impairment. While individuals with and without cognitive impairment share some fall risk factors such as history of falls, balance and gait impairments, physical deficits, reduced lower-limb strength, among others, there may be unique mechanisms and fall risk factors that are only present in cognitively impaired adults. Potential reasons include more severe gait and balance impairments in this group, unresponsiveness to fall prevention programs, or specific cognitive impairment-related factors contributing to fall risk [10]. Previous reviews which have focused on cognitively impaired individuals have included more mixed populations (including many types of CI) and settings (mainly institutionalized facilities such as hospitals and long-term care homes [3, 14]) or have only evaluated specific intervention types such as physical activity [15, 16]. There are limited reviews which include meta-analyses on interventions to specifically help adults with cognitive impairment in a community-based setting, despite these people having a disproportionately high rate of falls and fractures and poorer outcomes after falls [17, 18].

Based on practical work with relevant stakeholders, including members of the LOOP Fall Prevention Community of Practice (a group of over 1000 members across Canada working in health care, rehabilitation, public health, research, government, and non-governmental organizations to inform, share ideas and support each other to improve the implementation of evidence-informed fall prevention practices) [19] it has also become clear that there is a need to better translate research into practice. Stakeholders, including practitioners, are requesting evidence for interventions and best practices to implement in order to reduce falls in adults with cognitive impairment who are living in the community. While there are existing fall prevention guidelines which consist of multifactorial assessment and inventions such as strength and balance retraining, home hazard assessment and intervention, vision assessment, and medication review [20], they are not specific to community-dwelling and/or cognitively impaired adults. As a result of insufficient and unclear evidence, these fall prevention guidelines do not provide any recommendations for the population with cognitive impairment. Stakeholders are struggling to find this information and are in need of actionable, evidence-based guidelines and recommendations for practice.

Our review will directly address these gaps in evidence and practice by identifying the effectiveness of primary and secondary fall prevention interventions in community-dwelling adults with mild to moderate cognitive impairment. This will support future work which builds upon gaps in the research and addressing the barriers and facilitators of implementing fall prevention activities in the community [8, 9]. The outcomes of this review may have implications for research, practice, and policy.

## Methods

This systematic review and meta-analysis followed the Preferred Reporting Items for Systematic Reviews and Meta-analyses (PRISMA) guidelines [21] and reports on the outcomes ranked critical by our interdisciplinary research team (H.G., S.W.H., M.M.R., J.P., L.H., R.S., D.S.) from a registered protocol (PROSPERO-CRD42020210916). The protocol was also reviewed by three experts in the field of fall prevention and/or cognitive impairment. Our methods follow the Cochrane Handbook for Systematic Reviews of Interventions Version 6, 2019 [22].

### Search strategy

The search terms, databases, and strategy were developed in consultation with a research librarian at McMaster University and informed by previous systematic reviews [13, 14, 18] (Additional file 1). We searched MEDLINE, Embase, PsycINFO, Cochrane Central Register of Controlled Trials (CENTRAL), Cumulative Index of Nursing and Allied Health Literature (CINAHL), Web of Science, and Science Direct up to April 2020 and manually searched reference lists of relevant reviews and included studies for citations not captured in our search. Results from the search were deduplicated, and citations were uploaded to a secure internet-based platform for screening (DistillerSR, Evidence Partners Inc., Ottawa, Canada).

### Eligibility criteria

We included fall prevention interventions in community-dwelling adults (aged 50+) with mild and/or moderate CI. CI had to be assessed by a valid and reliable tool, diagnosis or medical report, and/or clearly identified and described by study authors. Studies with general adult populations or mixed populations but which have subgroup analysis for participants with mild or moderate cognitive impairment, were also considered. Without subgroup analysis, a mixed population must have at least 80% of participants with our targeted condition (mild to moderate cognitive impairment) to be included in our review. For our review, community dwelling included individuals living in a community setting (with or without caregiver support) and can include different locations/settings, however, we excluded those living in retirement homes, nursing homes, long-term care homes, acute care, or hospital settings where they may receive full-time support and care for activities of daily living. We also excluded studies of older adults with severe CI.

The main purpose of the intervention had to be either primary or secondary fall prevention as defined by the Institute for Work and Health [23]. Briefly, primary fall prevention aims to prevent a fall before it ever occurs whereas secondary fall prevention aims to reduce the number and severity of falls in those who have already experienced falls. Studies must have been available in English, peer-reviewed, and comprised of interventions with a control group (randomization was not required). For our review, a control group was defined as treatment as usual, usual care (i.e., no change to usual activities), or minimal contact (an intervention not thought to reduce falls such as general health education or social visits).

Outcomes were not used for inclusion or exclusion of studies. Outcomes of interest were selected by our interdisciplinary research team (H.G., S.W.H., M.M.R., J.P., L.H., R.S., D.S.) through an anonymized voting process. This process involved gathering a comprehensive list of outcomes and their associated tools/measurements based on our research and clinical expertise, factors related to falls risk [4], and existing and relevant systematic reviews [13, 14, 18]. The team identified any missing outcomes and then anonymously ranked the outcomes on a scale of 1–9 (< 4 not important, 4–6 important, 7–9 critical) based on Grading of Recommendation, Assessment, Development and Evaluations (GRADE) methodology [24]. Those not involved in the ranking process (D.F.L., M.R.) compiled and averaged the scores for each outcome and provided the list of outcomes to the committee for final discussion and agreement. Outcomes with an average score that reached critical were included in this review and consisted of Falls, Perceived Risk of Falls/Fear or Concern of Falls, Balance, Other fall measures (such as slips and trips), Gait Speed and Control, Functional Mobility, and Mortality. The full list of outcomes, their definitions, and the tools used to measure these outcomes can be found in Additional file 2.

### Data extraction and quality assessment

A team of researchers conducted the screening and data extraction (M.R., D.F.L., R.L., M.J.). A minimum of two reviewers were required to independently and in duplicate screen titles and abstracts of all potentially eligible studies. Articles marked for inclusion by any team member went on to full-text screening which was completed independently and in duplicate by 2 team members and required consensus for inclusion or exclusion. We developed, piloted, and deployed standardized forms for data extraction. Two team members independently completed full data extraction of study characteristics (setting, sample size, inclusion and exclusion criteria, characteristics of participants, type of intervention (categories based on existing literature [25] and taxonomies [26]), and experimental and control components) and intention-to-treat data for the outcomes listed above. In cases where studies had multiple measures for the same outcome, we extracted the primary or direct measures before using secondary outcomes or subgroup analysis data (i.e., within the same study, gait speed was preferred over dual-task cost measures). Reviewers also assessed study risk of bias (RoB) using the Cochrane Collaboration RoB tool [27] for randomized controlled trials (RCTs). All extraction was independently verified by the statistician (M.A.). Conflicts were resolved by the lead researcher of this review (M.R.).

We independently evaluated the certainty of the body of evidence using the Grading of Recommendation, Assessment, Development and Evaluations (GRADE) method [28] with GRADEpro software [29]. GRADE rates the certainty of a body of evidence as high, moderate, low, or very low and ratings are based on an assessment of 5 conditions: 1. methodological quality, 2. consistency across effect estimates/statistical heterogeneity, 3. directness of the body of evidence to the populations, interventions, comparators and/or outcomes of interest, 4. precision of results, and 5. indications of reporting bias.

### Statistical analysis

All data analyses were planned a priori. A meta-analysis was used to combine the results across studies by outcome using the published data from included studies. For the binary outcomes, we utilized the number of events; proportion or percentage data to generate the summary measures of effect in the form of risk ratio (RR) using DerSimonian and Laird random effects models with inverse variance method [30].

For continuous outcomes, we used immediate post-treatment data (means, standard deviations of change from baseline scores). The DerSimonian and Laird random effects models with inverse variance (IV) method were used to generate the summary measures of effect in the form of mean difference (MD) [30]. For instances where multiple outcome measures were used for the same outcome, we generated the summary measures of effect in the form of standardized mean differences (SMD) [30]. The SMD was used as a summary statistic because the studies in this systematic review often assessed the same outcome measured in a variety of ways (e.g., gait measured as gait speed, stride length, stride time, 6 m walk test (6MWT), use of gait aids, step tests, Dynamic Gait Index (DGI), coefficient of variation, etc.). In this situation, it was necessary to standardize the results of the studies before they could be compared across studies or combined in a quantitative synthesis. The SMD based effect sizes represent the magnitude of intervention effect relative to the variability observed within a particular study. Therefore, the studies for which the difference in mean change score was the same as the proportion of standard deviation of mean change score will have the same SMD, regardless of the actual scale or unit of measurement used to assess the outcome measures [31, 32]. The SMD is interpreted based on its magnitude according to Cohen d recommended thresholds (~ 0.2 = small effect, ~ 0.5 = medium effect, ~ 0.8 = large effect) [33]. For studies where measure of variance was reported as confidence intervals, standard error, or *p*-values, we used Cochrane recommended methods to convert this data to standard deviation [31].

We used a random effects multi-level meta-analytic approach to account for dependency between effect sizes (i.e., the correlation between effect sizes due to multiple measures or sub-measures of the same outcome within a study or comparison of multiple interventions to a single control group). In such cases, multiple measures and comparisons from the same study were nested within studies first and variance in observed effect sizes was decomposed into sampling variance, within study variance and between-study variance to account for intracluster (or intraclass) correlation in the true effects [32, 34]. For pooling of performance measures, the direction of effect was adjusted to ensure consistency of desirable outcome responses (i.e., reduction in gait speed measured in seconds to cover a standard distance reflects a better outcome, whereas an increase in gait speed measured in meters per second reflects a positive outcome).

We conducted further subgroup and meta-regression analysis based on fall prevention intervention type and study quality (risk of bias) where possible (for outcomes where there were more than 5 studies).

The Cochran’s Q (α = 0.05) was employed to detect statistical heterogeneity and I^2^ statistic to quantify the magnitude of statistical heterogeneity between studies where I^2^ > 50% represents moderate and I^2^ > 75% represents substantial heterogeneity across studies [32, 34]. The statistical heterogeneity I^2^ statistic was estimated in the context of multi-level meta-analytical approach i.e. within-cluster heterogeneity (i.e. across effect sizes or multiple arms from same study) and between-cluster heterogeneity (i.e. effect sizes across studies or subgroups of interest) [32, 34]. Overall I^2^ for each summary effect size was estimated to represent the heterogeneity not attributable to sample error and is the sum of within-cluster and between-cluster heterogeneity. All analyses were performed using R software (metafor [35] and dmetar [36] packages).

## Results

**Fig. 1:**
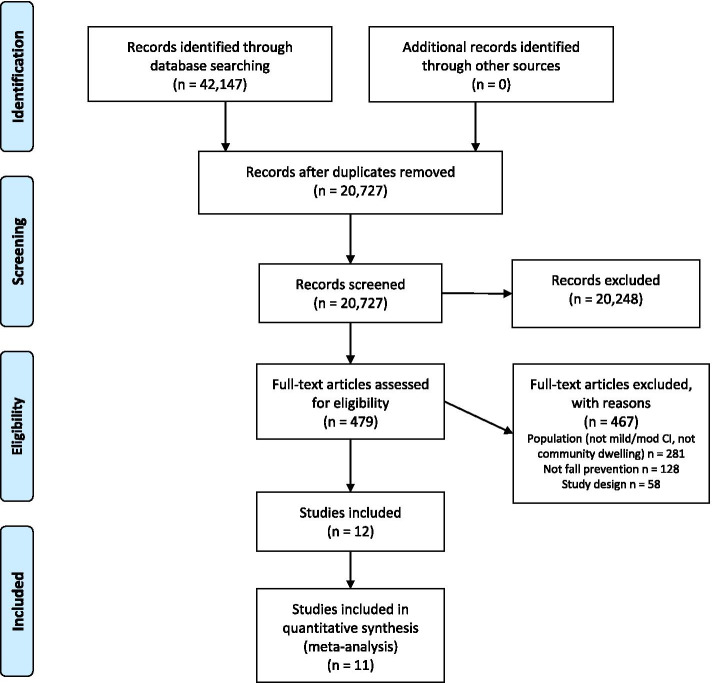
PRISMA Flowchart

From 20,727 citations, we assessed 479 full-text articles for eligibility, and included 10 RCTs and 2 clinical controlled trials (CCTs) in this review (Fig. 1) [37]. The majority of studies (*n* = 8) were exercise interventions [37–44], while 3 were multifactorial [17, 45, 46], and 1 provided medication treatment [47]. All studies included one intervention/treatment group and one control group, except for one study which tested two versions of the same intervention at different intensities (high or moderate intensity) compared to one control group [38]. The studies were published from 2010 to 2020. Further details of the included studies can be found in Table 1 and demographic data from studies can be found in Additional file 3. A total sample of 509 community-dwelling adults with mild to moderate cognitive impairment were included in this review with a mean age ranging from 67.5 to 84.0 years and percentage of women in the studies ranging from 20 to 74%. All included studies had fewer than 122 participants with most studies consisting of less than 50 participants total (*n* = 8). Attrition rates ranged greatly from 4.5% [17] to 71.4% [37]; however, most studies had attrition rates in the ranges of 9–20% (Additional file 3). Studies were conducted across the globe in North America, South America, Europe, Asia, and Australia, and intervention duration was between 4 weeks to 1 year, but most studies (*n* = 8) were between 12 weeks and 6 months in duration.

**Table 1:** Demographics and Characteristics of Fall Prevention Intervention Included Studies

Study, Year (ref)	Location	N^**1**^	Age, mean y (SD)	Gender^**2**^ (F/M, %)	Tool (score/cut off), Baseline Score, & Level of CI	Study Design	Intervention Duration^**3**^	Intervention Category & Setting	Control	Outcomes	Harms
Varriano, 2020 [37]	Canada	7	O: 79.1 (6.7)	57/43	MoCA (15–26) O: 21.2 (2.9) Mixed CI	RCT	12 weeks	Exercise; vestibular exercises N/R	Usual care	Balance, Gait speed and control	Falls, but unclear if due to intervention
Goldberg, 2019 [38]	United Kingdom	60	O: 76 (range 65–91)	43/57	MMSE (18–26) O: 25.6 (3.1); I: 24.8 (3.6); 26.2 (3.2); C: 25.9 (2.4) Mild dementia or CI	RCT	12 months	Exercise; Balance, strength, dual-task training, gait re-education Home-based	Single falls prevention assessment	Falls, Perceived risk of falling, Balance, Gait speed and control, Functional mobility (TUG)	19 recorded adverse events (5 non-serious but intervention related)
Padala, 2017 [39]	USA	30	O: 73.0 (6.2); I: 72.1 (5.3); C: 73.9 (7.1)	37/63	MMSE (≥18) O: 22.9 (2.2); I: 23.3 (2.2); C: 22.7 (2.3) Mild AD	RCT	8 weeks	Exercise; Wii-fit (yoga, strength, aerobics, balance) Home-based	Self-paced walking program	Perceive risk of falling, Balance	None study related
Zieschang, 2017 [40]	Germany	122	I: 82.1 (6.6); C: 82.2 (6.7)	74/26^2^	MMSE (17–26) I: 21.6 (2.9); C: 21.9 (3.3) Mild to moderate dementia	RCT	3 months	Exercise; progressive resistance and functional training (activities of daily living, balance, walking, gait) N/R	Seated motor training exercises	Falls	N/R
Sungkarat, 2017 [41]	Thailand	66	I: 68.3 (6.7); C: 67.5 (7.3)	50/50	MoCA (< 26), MMSE (≥24) I: MoCA: 21.2 (3.4), MMSE: 26.5 (1.7); C: MoCA: 20.4 (3.8), MMSE: 25.8 (2.3) Mild CI	RCT	15 weeks	Exercise; Tai Chi Community-centre and home-based	Educational material covering information related to cognitive impairment and fall prevention	Perceive risk of falling, Balance, Functional mobility (muscle strength data^*^)	No adverse events found.
Schwenk, 2016 [42]	USA	22	O: 78.2 (8.7); I: 77.8 (6.9); C: 79.0 (10.4)	55/45	MoCA (> 20) O: 23.3 (2.6); I: 23.3 (3.1); C: 22.4 (3.0) Mild CI	RCT	4 weeks	Exercise; Balance (ankle point-to-point reaching tasks and virtual obstacle-crossing tasks) Research centre	Usual care	Perceive risk of falling, Balance, Gait speed and control	No training-related adverse events occurred.
Montero-Odasso, 2019 [47]	Canada	60	O: 75.28 (7.18); I: 73.45 (5.74); C: 77.24 (8.11)	45/55	CDR (0.5), MMSE, MoCA O: sMMSE: 27.47 (1.96), MoCA: 23.60 (2.52); I: sMMSE: 27.42 (2.19), MoCA: 23.19 (2.55); C: sMMSE: 27.52 (1.72), MoCA: 22.97 (2.37) Mild CI	RCT	6 months	Medication or vitamin supplement; Donepezil Home-based	Placebo	Falls, Balance, Gait speed and control	No major adverse events requiring treatment were reported.
Chen, 2018 [45]	Taiwan	30	I: 77.3 (9.4); C: 77.3 (10.0)	50/50	MMSE, CDR (0.5, 1 or 2) I: MMSE: 16.4 (7.3), CDR: 0.5 = 6, 1.0 = 6, 2.0 = 3; C: MMSE: 17.9 (3.7), CDR: 0.5 = 3, 1.0 = 9, 2.0 = 1 Mild to moderate dementia	RCT	2 months	Multifactorial; Musical dual-task training (physical and cognitive tasks) Community/research centre	Non-musical cognitive tasks and walking exercises	Perceive risk of falling, Gait speed and control, Functional mobility (TUG)	No adverse events reported.
Kim, 2017 [46]	Korea	30	I: 82.0 (4.6); C: 80.9 (3.4)	20/80	MMSE-Korea I: 15.5 (2.9); C: 15.6 (2.4) Mild to moderate dementia	CCT	12 weeks	Multifactorial; physical activities, cognitive activities, activities of daily living, music activities Community centre	Usual care	Perceive risk of falling, Balance, Functional mobility (TUG & CST)	N/R
Wesson, 2013 [17]	Australia	22	I: 78.7 (4.2); C: 80.9 (5.0)	41/59	ACE-R (≤82), MMSE I: ACE-R: 67.8 (12.6), MMSE: 24.5 (3.1); C: ACE-R: 62.5 (14.2), MMSE: 22.5 (4.3) Mild dementia	RCT	12 weeks	Multifactorial; strength and balance exercises, home hazard reduction Home-based	Usual care, health promotion brochures on fall prevention and home safety	Falls, Perceive risk of falling, Balance, Gait speed and control	No serious adverse events related to the intervention were reported. Minor complaints relating to stiffness, dizziness and mild joint pain (*n* = 4; 36%) were reported.
Suttanon, 2013 [43]	Australia	40	O: 81.90 (5.72); I: 83.42 (5.10); C: 80.52 (6.01)	63/37	MMSE (≥10) I: 20.89 (4.74); C: 21.67 (4.43) Mild to moderate AD	RCT	6 months	Exercise; balance and strength exercises, walking program Home-based	Education and information sessions on the topic of dementia and ageing	Falls, Perceive risk of falling, Balance, Gait speed and control, Functional mobility (TUG, CST, and FRT^*^)	There were no falls or other serious adverse events associated with the intervention
Hernandez, 2010 [44]	Brazil	20	O: 78.5 (6.8); I: 77.7 (7.6); C: 84.0 (6.1)	N/R	CDR; MMSE I: 16.4 (6.7); C: 14.2 (5.1) Mild to moderate AD	CCT	6 months	Exercise; stretching, weight training, circuits, dance, recreational activities, relaxation N/R	Usual care	Balance, Functional mobility (TUG)	N/R

*CI* cognitive impairment, *O* overall population, *I* intervention, *C* control, *AD* Alzheimer’s Disease, *N/R* not reported, *RCT* randomized controlled trial, *CCT* clinical (non-randomized) controlled trial. *MoCA* Montreal cognitive assessment (Score /30), *MMSE* Mini Mental State Exam, *ACE-R* Addenbrooke’s cognitive examination – revised, *CDR* Clinical Dementia Rating scale, *TUG* timed up and go test, *CST* chair sit stand test, *FRT* functional reach test. ^1^ Number of participants randomized to intervention; ^2^ Values for gender are based on reported baseline which may not equal N randomized but rather the number of participants who completed the intervention; ^3^ Not including follow-up, if applicable; ^*^outcome not meta-analyzed

The majority of studies (*n* = 10) used the Mini Mental State Exam (MMSE), or some variation, for assessing their participants’ cognitive impairment. Four studies used the Montreal Cognitive Assessment (MoCA) tool. These tools were also used for inclusion of participants based on specified cut-off scores, which varied depending on the study. Formal diagnosis of mild to moderate cognitive impairment, dementia, or Alzheimer’s Disease by a physician or qualified standard such as Petersen’s criteria [48] or the DSM-IV: Diagnostic and Statistical Manual of Mental Disorders [49], was also considered for inclusion of participants in 11 of the studies. Seven of the included studies specifically stated there were no serious adverse events related to the intervention, 3 made no mention of adverse events, and 3 reported some minor adverse events due to the intervention such as stiffness, mild joint pain, or were unclear if the adverse events were intervention-related.

### Risk of Bias and quality of included studies

The Cochrane RoB tool showed mixed quality of study methodology: 2 studies were low risk of bias [39, 41], 3 studies were high risk of bias [42, 44, 45], and 7 studies were rated as an unclear risk of bias [17, 37, 38, 40, 43, 46, 47], mostly due to issues around allocation concealment, blinding, and incomplete outcome data (Table 2).

**Table 2:**
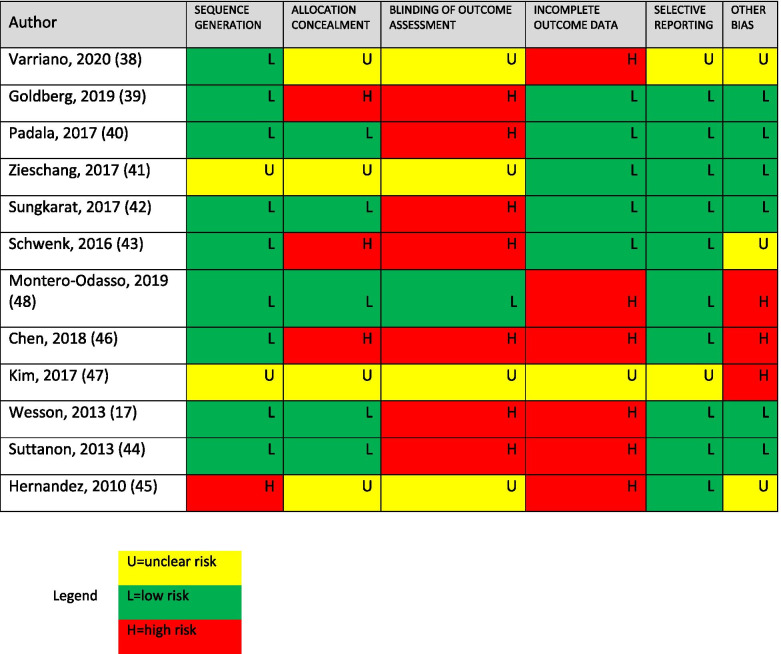
Risk of Bias for Included Studies

The certainty of evidence, as assessed by GRADE, ranged from very low to moderate but was moderate for most outcomes (*n* = 4) due to downgrading for risk of bias concerns (Table 3; full GRADE tables by outcome can be found in Additional file 4).

**Table 3:** Benefits of Treatment; Results of Meta-Analysis by Outcome for Included Studies (*n* = 12)

Outcome	# studies | N	SMD (95% Confidence interval)	GRADE rating
Falls (# events)	4 | 224	RR 0.99 (0.60, 1.65)	LOW downgraded for risk of bias and imprecision
Falls (incidence)	4 | 209	IR 0.90 (0.47, 1.71)	LOW downgraded for risk of bias and imprecision
**Perceived Risk of Falls / Fear of Falling**	**8 | 263**	**Medium; −0.73 (−1.10, − 0.36)**	**MODERATE** downgraded for risk of bias
**Balance**	**9 | 318**	**Medium; 0.66 (0.19, 1.12)**	**MODERATE** downgraded for risk of bias
**Gait speed and control**	**6 | 194**	**Small; 0.26 (0.08, 0.43)**	**MODERATE** downgraded for risk of bias
**TUG**	**5 | 151**	**Medium; −0.56 (−0.94, − 0.17)**	**MODERATE** downgraded for risk of bias
CST	2 | 70	No effect; 0.34 (−1.73, 1.06)	VERY LOW downgraded for risk of bias and imprecision

Bold denotes significance *p* < 0.05; *N* = total number of participants; *SMD* standardized mean difference, *RR* risk ratio, *IR* incidence rate, *TUG* timed up and go test, *CST* chair sit and stand test

### Benefits of treatment

We conducted a meta-analysis for all outcomes on 11 of the included studies. One study [37] was excluded from the meta-analysis and is only described qualitatively because of severe risk of bias concerns and the loss of all participants but 1 in each group during follow-up. Additionally, of the potential functional mobility outcome measures, our included studies only had data for the Chair Sit and Stand Test (CST) and the Timed Up and Go Test (TUG). Therefore, we analyzed these measures separately.

A number of different measures were used across the included studies for the various outcomes (Additional file 5). Falls as a direct outcome was measured by 5 different studies as numbers of falls (events) (*n* = 4) or incidence of falls (time to event) (*n* = 4) using self-reported falls calendars. Perceived risk of falls or fear of falls was measured by 8 studies using the Falls Efficacy Scale by 6 of the studies with this outcome. Other common measures included the Falls Risk for Older People or Physiological Profile Assessment. Balance was the most frequently reported outcome as it was measured by 9 different studies and included measurements such as the Berg Balance Scale, Postural Sway Tests, and Limits of Stability. Gait speed and control were reported by 6 studies and included a variety of gait speed measurements (habitual walking speed, fast walking speed), step tests and walk tests, Dual-task gait cost tests, and gait coefficient of variation. Lastly, functional mobility outcomes were assessed by either TUG and/or CST. Five studies assessed TUG with some variations in this test such as including cognitive or motor tasks. Only 2 studies assessed CST or sit to stand measurements. Our review first presents results of all the included fall prevention intervention studies on each outcome and then the subgroup analysis by intervention type and risk of bias for outcomes which had more than 5 studies.

#### Overall fall prevention interventions for all outcomes

**Fig. 2:**
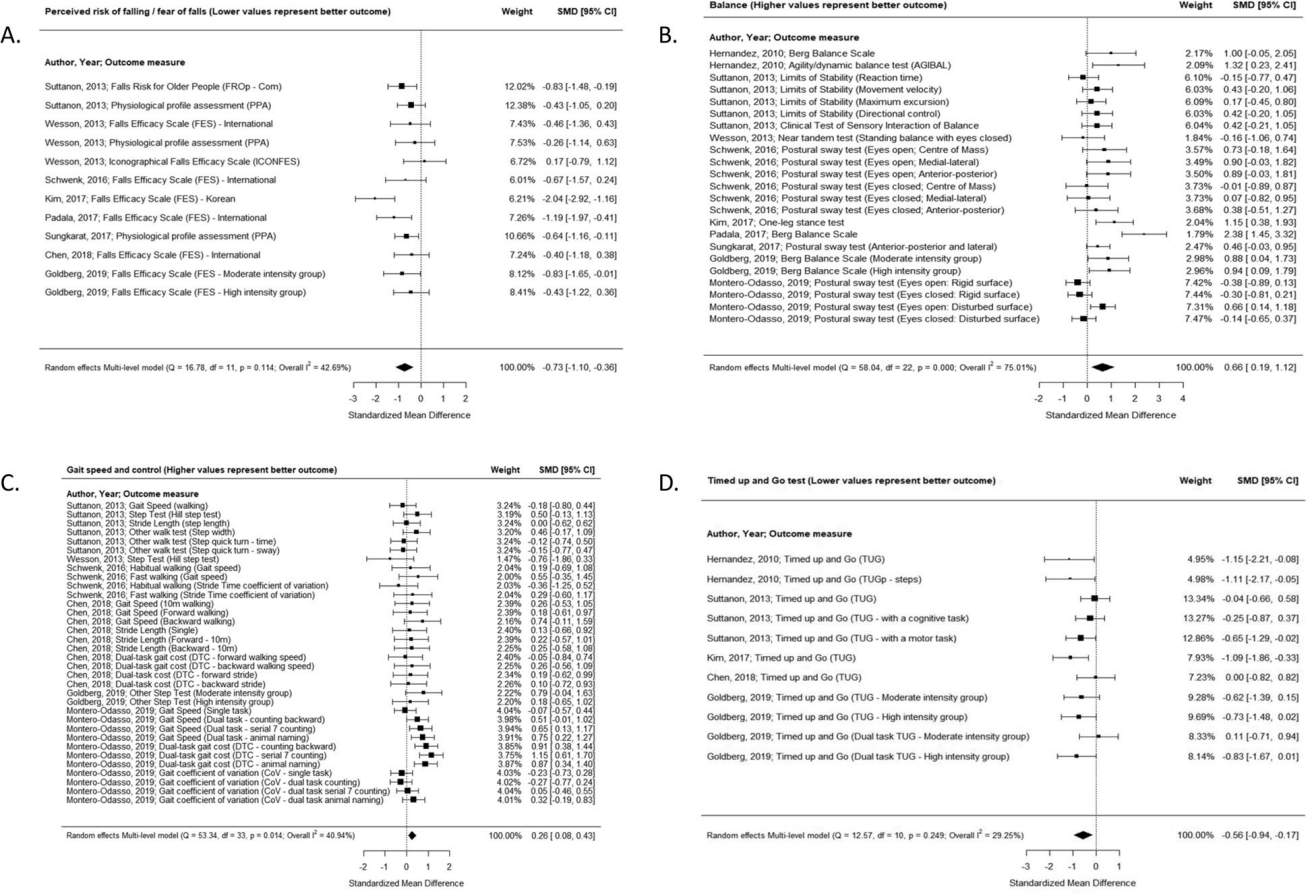
Effects of fall prevention interventions on perceived risk of falls (**A**), balance (**B**), gait speed and control (**C**), and timed up and go (**D**) outcomes. Weights are from random effects multi-level model analysis. Note: SMD = standardized mean difference, CI = confidence interval

Overall, fall prevention interventions had significant effects of medium magnitude on perceived risk of falls/fear of falling (SMD -0.73 [− 1.10, − 0.36]), balance (SMD 0.66 [0.19, 1.12]), and functional mobility as measured by TUG (SMD -0.56 [− 0.94, − 0.17]) and significant effects of small magnitude on gait speed and control (SMD 0.26 [0.08, 0.43]) all with moderate certainty of evidence (Fig. 2; Table 3). There were no significant effects for falls (number of events or falls incidence) and chair sit stand test outcomes (Table 3).

We were able to conduct subgroup analysis by intervention type and risk of bias for perceived risk of falls/fear of falling, balance, and gait speed and control as these outcomes had more than 5 studies with data (Table 4; Additional file 6).

**Table 4:** Results of Subgroup Analysis

Outcome Subgroup Analysis	# studies	SMD (95% CI)	*p*-value for subgroup difference*
Balance			
INTERVENTION TYPE			0.44
**Exercise**	6	**0.85 (0.26, 1.43)**	
Medication	1	−0.04 (− 0.80, 0.71)	
Multifactorial	2	0.52 (−0.77, 1.80)	
RISK OF BIAS			0.35
**Low**	2	**1.28 (0.20, 2.36)**	
Unclear	5	0.40 (−0.24, 1.04)	
High	2	0.77 (−0.22, 1.77)	
Perceived Risk of Falls			
INTERVENTION TYPE			0.93
**Exercise**	5	**−0.70 (− 1.02, − 0.37)**	
Multifactorial	3	−0.84 (− 2.42, 0.73)	
RISK OF BIAS			0.84
**Low**	2	**−0.88 (− 1.67, − 0.08)**	
**Unclear**	4	**−0.77 (− 1.32, − 0.22)**	
High	2	− 0.53 (− 1.41, 0.36)	
Gait Speed and Control			
INTERVENTION TYPE			0.27
Exercise	3	0.15 (−0.08, 0.38)	
**Medication**	1	**0.42 (0.09, 0.75)**	
Multifactorial	2	−0.12 (−1.17, 0.93)	
RISK OF BIAS			0.75
**Unclear**	4	**0.27 (0.03, 0.51)**	
High	2	0.21 (−0.12, 0.53)	

*for meta-regression test for differences between groups

BOLD denotes summary subgroup estimates that are statistically significant with *p* < 0.05

*SMD* standardized mean difference, *CI* confidence interval

#### Intervention type

Subgroup analysis by intervention type showed the benefit remained significant in exercise interventions for balance (SMD 0.85 [0.26, 1.43]), perceived risk of falls (SMD -0.70 [− 1.02, − 0.32]). The benefit also remained significant in the one medication intervention that measured gait speed and control (SMD 0.42 [0.09, 0.75]). However, the difference between intervention subgroups was not statistically significant for any of these outcomes (Table 4).

#### Risk of Bias

Subgroup analysis by risk of bias showed that low risk of bias studies remained significant for balance (SMD 1.28 [0.20, 2.36]) and perceived risk of falls (SMD -0.88 [− 1.67, − 0.08]). The benefit also remained significant in unclear risk of bias studies for perceived risk of falls (SMD -0.77 [− 1.32, − 0.22)] and gait control and stability (SMD 0.27 [0.03, 0.51]). However, the difference between risk of bias subgroups was not statistically significant for these outcomes (Table 4).

## Discussion

The purpose of this review was to evaluate the available evidence on the effectiveness of primary and secondary fall prevention interventions in community-dwelling adults with mild to moderate cognitive impairment. The effectiveness of the 12 included RCTs/CCTs was assessed based on six outcomes which were ranked by our interdisciplinary research team and included falls, perceived risk of falls/fear or concern of falls, balance, gait speed and control, and the two functional mobility outcomes of TUG and CST. The meta-analysis demonstrated that fall prevention interventions could be effective for decreasing participants’ perceived risk of falls/fear of falling (k = 8; *n* = 209) and improving their balance (k = 9; *n* = 318), gait speed and control (k = 6; *n* = 194), and TUG (k = 5; *n* = 151) based on moderate certainty of evidence. But there were no significant effects for falls (number of events (k = 4; *n* = 124) or falls incidence (k = 4; *n* = 209)) and chair sit stand test (k = 2; *n* = 70) outcomes. The subgroup analysis of our review also demonstrated that exercise interventions and high quality studies at lower risk of bias maintained these benefits, but not significantly more than other interventions or studies at a higher risk of bias. However, caution should be taken when interpreting the findings of our systematic review due to the small number of studies, small sample sizes in the included studies, and the heterogeneity of interventions and participants in the studies.

### Comparison with existing literature

While the body of evidence for fall prevention continues to grow, there are limited studies and reviews which include meta-analyses on interventions to specifically help adults with cognitive impairment in a community-based setting, despite these people having a disproportionately high rate of falls and fractures and poorer outcomes after falls [17, 18]. Published reviews are either focused on a general older adult population who are cognitively healthy [13, 50], on specific intervention types such as physical activity [15, 16, 51], or have focused on more mixed populations (including many types of CI) and settings [3, 12, 14]. In cognitively healthy adults, the evidence is supportive of multifactorial interventions, many of which include exercise, in reducing and preventing falls by approximately 20% [12, 13], and a systematic review of 4 interventions in older people with dementia found a 32% reduced risk of being a faller after an exercise program [16]; however, while we also found that exercise programs were common among fall prevention interventions, we did not see significant effects on falls in the studies in our review regardless of intervention type.

Cognitive impairment is a well-recognized risk factor for falls due to a variety of reasons including a lack of insight into environmental dangers or tripping hazards, a failure to comply with safety or medical treatment, an increased state of confusion, and the role cognition plays in gait [3, 52]. However, a better understanding is required of fall risk factors in this population [52]. In cognitively healthy adults and older adults, there are over 400 risk factors associated with falls [4]. Our review chose to include numerous outcomes and measurements/tools used to assess these outcomes to allow for a robust body of evidence and assessment of the effectiveness of fall prevention interventions in this population. The outcomes which showed significance from our meta-analysis of included fall prevention studies aligns with short-form screening tools such as the Stopping Elderly Accidents, Deaths, and Injuries (STEADI) initiative and the associated 3-item fall screening questionnaire [53]. This brief screening tool simply asks if the individual has fallen in the past year (falls outcome), feels unsteady when standing or walking (balance and gait outcomes), and if they are worried about falling (perceived fear of falls outcome). While our review did not find statistically significant intervention-related improvements, the surrogate outcomes which are related to falls risk (such as fear of falls, balance, and gait) provide signals and good data that there could be improvements in falls themselves. It is encouraging to see trends in four outcomes from moderate certainty of evidence. However, it is important to note that these outcomes have varying reliability in individuals with cognitive impairment [54–57] and the falls outcome was downgraded for certainty of evidence because of methodological issues and concerns related to risk of bias and imprecision.

The downgrading of evidence speaks to another important finding with implications for future research in this area related to study design and study quality. The subgroup analysis of this review signalled that higher quality studies showed beneficial and significant effects in outcomes related to fall prevention including balance, perceived risk of falls, and gait control and stability. These results must still be interpreted with caution due to the limited number of studies in our review overall and within each subgroup (as is evident by the wide confidence intervals), the possibility for underpowered studies with small sample sizes, and the high degree of heterogeneity across these interventions (as is evident by the variety of exercise interventions). Many of the included studies did not indicate that falls was their primary outcome and overall, the interventions were very short in duration, which may mean these studies were not equipped to see significant effects in outcomes which take longer to change. For example, the two studies which were evaluated as low risk of bias [39, 41] were only 8 weeks and 15 weeks long, had small number of participants (*n* = 30 and *n* = 66), were both exercise interventions, and neither measured falls as an outcome. However, it is promising that the subgroup analysis demonstrated that across outcomes, lower risk of bias studies maintained their benefits, and also identifies the need for more robust, high quality research to ascertain the findings from our review.

Another criticism of previous fall prevention reviews in those with cognitive impairment is the heterogeneity of sample population [3] and the same is true in our review. While we had strict definitions and criteria for inclusion of studies based on population, we found a broad range of participants in terms of how they were classified and diagnosed and the tools and cut-off scores that were used to characterize mild and moderate cognitive impairment. The diversity in the included participants of the studies from this review may have also made it difficult to see significant changes in the outcomes of interest as clinicians have stated the importance of appropriate timing for intervening with this population based on their cognitive abilities. This heterogeneity also limited our ability to conduct any subgroup analysis according to cognitive level.

Exercise and multifactorial interventions are the most frequently reported fall prevention studies in those both with and without cognitive impairment [3]. Similar to previous reviews [3, 16], we also found that exercise interventions were effective at showing benefit for outcomes related to fall risk factors in individuals with cognitive impairment. However, the evidence was less convincing for multifactorial interventions and neither intervention type was statistically effective at reducing or preventing falls themselves nor were exercise interventions statistically better than multifactorial interventions. While these findings are similar to the results of reviews in adults without cognitive impairment [50], it speaks to the challenges and complex factors that affect factors related to falls and the uniqueness of a population with cognitive impairment. There may need to be intervention modifications for cognitively impaired adults such as including the engagement of carers, regular contact by trained professionals, a greater choice of exercise, among others [17, 43]. We also were unable to do any subgroup analysis or investigation to further elicit the intensities, duration, components, or combination of these factors which may be related to their effectiveness.

### Limitations

Although our search was comprehensive, we did not explicitly search grey literature. We did verify our included studies with those of other similar reviews and our results align with previous research in this area [14–16]. While we were consistent in our application of our inclusion criteria for mild to moderate cognitive impairment, we recognize that a limitation of this review will be how authors report and describe their study participant’s level of cognitive impairment. There was ambiguity in how authors defined and used cut offs, which speaks to issues within this field of study and the limitations of using screening tests for study purposes in the absence of a true clinical diagnosis. Lastly, while we did not observe any significant asymmetry across funnel plots for publication bias, studies were small and have risk of bias concerns.

### Implications for practice and research

The findings from our review make it difficult to make clear recommendations for practice. Research in falls prevention among community-dwelling adults is still largely conducted on individuals without cognitive impairment and our review shows the difficulties in translating this research into practice in patients with cognitive impairment. The greater fall risk for adults with cognitive impairment includes different underlying mechanisms and there may be unique risk factors that are not present in cognitively normal adults. The heterogeneity of study participants and intervention components does not help explain the findings or suggest best practices for clinical practice. Clinicians should continue to work with their patients to implement fall prevention activities such as exercise and look for improvements in fall risk factors such as perceived risk of falls, balance, gait, and TUG.

Our review also demonstrates that evidence is still lacking for fall prevention interventions in those with cognitive impairment and further highlights that the needs of clinicians are not being met, despite calls advocating for large-scale, high quality research [3]. We found small studies, that were short in duration, not powered to detect falls, and had risk of bias issues. Subgroup analysis indicated that we need more high quality research (low risk of bias studies) to ascertain the findings from our review.

## Conclusions

Practitioners frequently see exercise interventions in recommendations for fall prevention but are unsure what they can do to help their patients with cognitive impairment. Our review and meta-analysis confirm the difficulties and limitations in this field of research, which echoes what practitioners are saying. However, there is promising evidence which suggests that fall prevention interventions, particularly exercise programs of all kinds, can assist in the improvement of fall risk factors in this population. Further high quality research is needed with longer durations and appropriately powered sample sizes to determine the most appropriate falls prevention interventions for community-dwelling adults with cognitive impairment.

## Supplementary Information

**Additional file 1.**

**Additional file 2.**

**Additional file 3.**

**Additional file 4.**

**Additional file 5.**

**Additional file 6.**

## Data Availability

The main study data is the data extraction materials and quality ratings of included papers, most of which are included in the manuscript tables. Any other supporting data relating to this review is available from the authors.

## Abbreviations

ACE-R: Addenbrooke’s cognitive examination – revised
AD: Alzheimer’s Disease
ADLs: Activities of daily living
C: Control
CCT: Clinical controlled trial
CDR: Clinical dementia rating scale
CENTRAL: Cochrane Central Register of Controlled Trials
CI: Cognitive impairment
CINAHL: Cumulative Index of Nursing and Allied Health Literature
CoV: Coefficient of variation
CST: Chair sit stand test
DGI: Dynamic gait index
DSM-IV: Diagnostic and Statistical Manual of Mental Disorders
DTC: Dual-task cost
FES: Falls efficacy scale
FROp: Falls risk for older people
FRT: Functional reach test
GRADE: Grading of Recommendation, Assessment, Development and Evaluations
I: Intervention
ICONFES: Iconographical falls efficacy scale
IR: Incidence rate
IV: Inverse variance
MCI: Mild cognitive impairment
MD: Mean difference
MMSE: Mini Mental State Exam
MoCA: Montreal Cognitive Assessment
N: Total number of participants
N/R: Not reported
O: Overall population
PPA: Physiological profile assessment
PRISMA: Preferred Reporting Items for Systematic Reviews and Meta-analyses
RCT: Randomized controlled trial
RoB: Risk of bias
RR: Risk ratio
SMD: Standardized mean difference
STEADI: Stopping Elderly Accidents, Deaths, and Injuries
TUG: Timed up and go test
6MWT: 6 m walk test
