# Supplementary material for: Fall prevention in community-dwelling adults with mild to moderate cognitive impairment: a systematic review and meta-analysis
Source: BMC Geriatr. 2021 Dec 10;21:689. doi: 10.1186/s12877-021-02641-9 (PMC8665555; doi:10.1186/s12877-021-02641-9)
Supplement: Supplementary file 2 — Additional file 2. [file 12877_2021_2641_MOESM2_ESM.docx]

Additional File 2:

**Outcomes and Definitions for Data Extraction**

1. Actual falls
   1. Definition: Unintentionally coming to rest on the ground or floor or other lower level, regardless of whether the fall resulted in injury ([Registered Nurses Association of Ontario [RNAO], 2005](https://muse.jhu.edu/article/376778#b70)) or “an unexpected event in which the participants come to rest on the ground, floor, or lower level” (ProFANE Lamb et al 2005)
   2. Measured by: *Total falls, recurrent falls, injurious falls (post-fall fractures or those requiring medical attention), falls incidence, rate of falls (per hrs of activity, day, etc), number of fallers (people), time to first fall.**Often a falls calendar or self-reported falls measure
2. Perceived risk of falling/fear or concern of falls
   1. Definition: Fear of falling (FOF) refers to persistent concern about sustaining a fall that, in turn, causes an individual to avoid daily activities. FOF often becomes a barrier to walking and participation in physical activities, leading to an abnormal gait and a loss of confidence in walking ability. Persistent FOF has been associated with deconditioning, social isolation, depression, more frequent falls, greater frailty, decline in mobility and increased mortality (LOOP Fear of Falling report).
   2. Measured by: FROP-COM, falls risk for older people – community version, Falls Efficacy Scale – International (short form), Iconographical Falls Efficacy Scale (ICONFES), *physiological profile assessment (PPA)*
3. Balance
   1. Definition: Objectively measures of participant’s ability to maintain balance static or during the performance of various tasks or tests.
   2. Measured by: one-leg standing, Berg Balance Scale, limits of stability (movement velocity), Tandem, near tandem test of standing balance with eyes closed, postural sway tests, agility/ dynamic balance test (AGIBAL)
   3. NOT: dizziness handicap inventory (DHI)
4. Other fall measures (Slips, trips, or near falls)
   1. Definition of slips/trips: regaining balance without a fall ([Steinberg et al., 2000](https://muse.jhu.edu/article/376778#b83)); data are limited on the prevalence of slips and trips and the prevention of such incidents in this age group (Steinberg et al., 2000). Yet, the most common circumstances leading to falls are slips and trips. It has been proposed that there is a continuum from slips and trips, wherein balance is regained, through near falls, to complete falls when balance is lost (Steinberg et al., 2000).
   2. Measured by: self-report, falls calendar, Falls Surveillance Report
5. Gait speed and control
6. Definition: Measures and characteristics of gait dynamics and pattern of walking or other gait impairments. Measures of gait impairment are valid markers of mobility decline and falls risk.
7. *Measured by: gait speed, stride length, stride time, 6MWT, use of gait aids, step tests,* Dynamic Gait Index (DGI), coefficient of variation
8. Functional mobility
9. Definition: Person's physiological ability to move independently and safely in a variety of environments in order to accomplish functional activities or tasks and complete everyday tasks.
10. *Measured by: FIM, functional independence measure; SPPB, short physical performance battery; FR, functional reach; TUG, timed up and go; CST, Chair sit stand test; POMA; Muscle strength (lower limb muscle strength))*
11. Mortality
12. Mortality of study participant that is falls related or any cause
13. Measured by: Any report by researchers of death of participant
