# Supplementary material for: Fall prevention in community-dwelling adults with mild to moderate cognitive impairment: a systematic review and meta-analysis
Source: BMC Geriatr. 2021 Dec 10;21:689. doi: 10.1186/s12877-021-02641-9 (PMC8665555; doi:10.1186/s12877-021-02641-9)
Supplement: Supplementary file 4 — Additional file 4. [file 12877_2021_2641_MOESM4_ESM.docx]

**Additional File 4:**

**GRADE evidence ratings:** Targeted falls prevention interventions compared to usual care for Community-dwelling adults (aged 50+) with mild or moderate cognitive impairment

| **Certainty assessment** | | | | | | | **№ of patients** | | **Effect** | | **Certainty** | **Importance** |
| --- | --- | --- | --- | --- | --- | --- | --- | --- | --- | --- | --- | --- |
| **№ of studies** | **Study design** | **Risk of bias** | **Inconsistency** | **Indirectness** | **Imprecision** | **Other considerations** | **Targeted falls prevention** | **Usual care** | **Relative / Absolute (95% CI)** | |  |  |
| **Risk of falls (follow up: range 12 weeks to 12 months; assessed with: Number of events/participants experiencing at least 1 fall)** | | | | | | | | | | | | |
| 4 ^a^ | randomised trials | serious ^b^ | not serious ^c^ | not serious | serious ^d^ | none ^e^ | 45/111 (40.5%) | 44/113 (38.9%) | **RR 0.99** (0.60 to 1.65) | **4 fewer per 1,000** (from 156 fewer to 253 more) | ⨁⨁◯◯ LOW | CRITICAL |
| **Incidence of Falls (follow up: range 12 weeks to 12 months; assessed with: Incidence rate – time to event)** | | | | | | | | | | | | |
| 4 ^f^ | randomised trials | serious ^g^ | not serious ^h^ | not serious | serious ^d^ | none ^e^ | 111 | 98 | **Incidence Rate ratio 0.90** (0.47 to 1.71) | | ⨁⨁◯◯ LOW | CRITICAL |
| **Perceived risk of falling (follow up: range 12 weeks to 12 months; assessed with: Continuous measures / tools)** | | | | | | | | | | | | |
| 8 ^i^ | randomised trials | serious ^j^ | not serious ^c^ | not serious | not serious ^k^ | none ^e^ | 139 | 124 | SMD **0.73 SD lower** (1.1 lower to 0.36 lower) | | ⨁⨁⨁◯ MODERATE | CRITICAL |
| **Balance (follow up: range 4 weeks to 12 months; assessed with: Continuous measures / tools)** | | | | | | | | | | | | |
| 9 ^l^ | randomised trials | serious ^m^ | not serious ^n^ | not serious | not serious ^k^ | none ^e^ | 168 | 150 | SMD **0.66 SD higher** (0.19 higher to 1.12 higher) | | ⨁⨁⨁◯ MODERATE | CRITICAL |
| **Gait control and stability (follow up: range 4 weeks to 12 months; assessed with: Continuous measures / tools)** | | | | | | | | | | | | |
| 6 ^o^ | randomised trials | serious ^p^ | not serious ^q^ | not serious | not serious ^k^ | none ^e^ | 105 | 89 | SMD **0.26 SD higher** (0.08 higher to 0.43 higher) | | ⨁⨁⨁◯ MODERATE | CRITICAL |
| **Timed up and go (TUG) (follow up: range 5 weeks to 12 months; assessed with: Timed up and go test)** | | | | | | | | | | | | |
| 5 ^r^ | randomised trials | serious ^s^ | not serious ^q^ | not serious | not serious ^k^ | none ^e^ | 83 | 68 | SMD **0.56 SD lower** (0.94 lower to 0.17 lower) | | ⨁⨁⨁◯ MODERATE | CRITICAL |
| **Chair sit to stand (CST) (follow up: range 5 weeks to 6 months; assessed with: Chair sit to stand test )** | | | | | | | | | | | | |
| 2 ^t^ | randomised trials | serious ^b^ | not serious ^n^ | not serious | very serious ^u^ | none ^e^ | 34 | 36 | SMD **0.34 SD lower** (1.73 lower to 1.06 higher) | | ⨁◯◯◯ VERY LOW | CRITICAL |

**CI:** Confidence interval; **RR:** Risk ratio; **SMD:** Standardised mean difference

#### Explanations

a. Suttanon, 2013; Wesson, 2013; Zieschang, 2017; Montero-Odasso, 2019

b. Studies were rated as unclear risk with serious concerns regarding blinding, incomplete outcome reporting and other risk of bias (i.e. baseline imbalance between groups, industry funding etc.).

c. The confidence intervals overlap with moderate statistical heterogeneity observed across studies.

d. The sample size is not adequate (<300) in each arm and effect estimate is imprecise with confidence intervals including the no effect value of "1".

e. Too few studies (n<10) to access publication bias.

f. Suttanon, 2013; Wesson, 2013; Zieschang, 2017; Goldberg, 2019

g. Studies were rated as unclear risk with serious concerns regarding allocation concealment, blinding, and incomplete outcome reporting.

h. The confidence intervals overlap with minimal statistical heterogeneity observed across studies.

i. Suttanon, 2013; Wesson, 2013; Schwenk, 2016; Kim, 2017; Padala, 2017; Sungkarat, 2017; Chen, 2018; Goldberg, 2019

j. Two studies were rated as high risk and four studies were rated as unclear risk with serious concerns regarding allocation concealment, blinding, incomplete outcome reporting and other risk of bias (i.e. baseline imbalance between groups, industry funding etc.).

k. The sample size is not adequate (<300) in each arm, however, effect estimate is precise with confidence intervals not including the no effect value of "0".

l. Hernandez, 2010; Suttanon, 2013; Wesson, 2013; Schwenk, 2016; Kim, 2017; Padala, 2017; Sungkarat, 2017; Goldberg, 2019; Montero-Odasso, 2019

m. Two studies were rated as high risk and five studies were rated as unclear risk with serious concerns regarding randomization, allocation concealment, blinding, incomplete outcome reporting and other risk of bias (i.e. baseline imbalance between groups, industry funding etc.).

n. High statistical heterogeneity observed, however, the direction of effect is consistent across most studies with overlapping confidence intervals and statistical heterogeneity is likely due to small versus large effects observed across studies.

o. Suttanon, 2013; Wesson, 2013; Schwenk, 2016; Chen, 2018; Goldberg, 2019; Montero-Odasso, 2019

p. Two studies were rated as high risk and four studies were rated as unclear risk with serious concerns regarding randomization, allocation concealment, blinding, incomplete outcome reporting and other risk of bias (i.e. baseline imbalance between groups, industry funding etc.).

q. Moderate statistical heterogeneity observed, however, the direction of effect is consistent across most studies with overlapping confidence intervals and statistical heterogeneity is likely due to small versus large effects observed across studies.

r. Hernandez, 2010; Suttanon, 2013; Kim, 2017; Chen, 2018; Goldberg, 2019

s. Two studies were rated as high risk and three studies were rated as unclear risk with serious concerns regarding randomization, allocation concealment, blinding, incomplete outcome reporting and other risk of bias (i.e. baseline imbalance between groups, industry funding etc.).

t. Suttanon, 2013; Kim, 2017

u. The sample size is not adequate (<300) in each arm and effect estimate is imprecise with confidence intervals including the no effect value of "0".
