# Supplementary material for: Fall prevention in community-dwelling adults with mild to moderate cognitive impairment: a systematic review and meta-analysis
Source: BMC Geriatr. 2021 Dec 10;21:689. doi: 10.1186/s12877-021-02641-9 (PMC8665555; doi:10.1186/s12877-021-02641-9)

**Additional File 5: Forest Plots for all Meta-Analyzed Outcomes**

**Metanalysis: Targeted falls prevention interventions compared to usual care for Community-dwelling adults (aged 50+) with mild or moderate cognitive impairment**


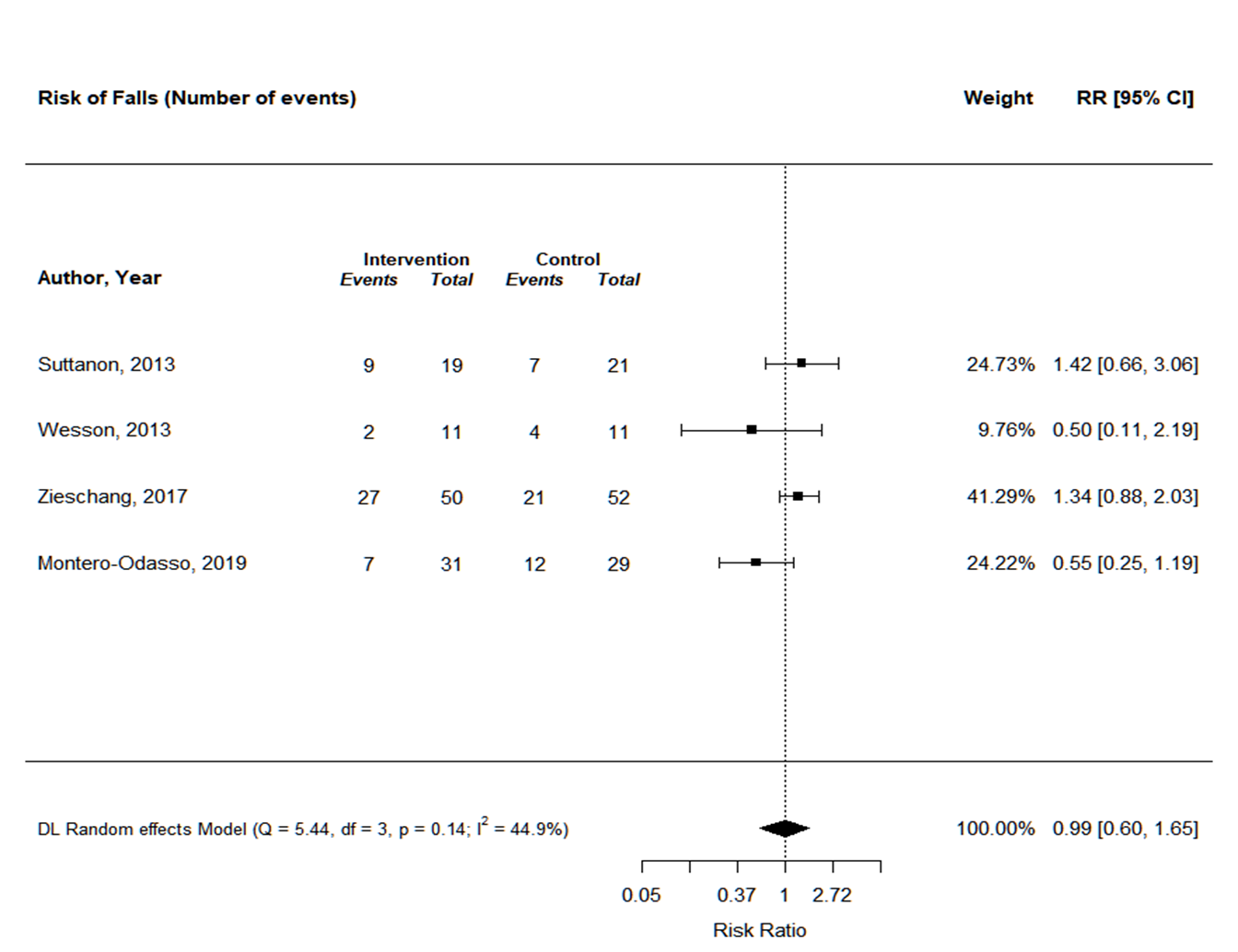


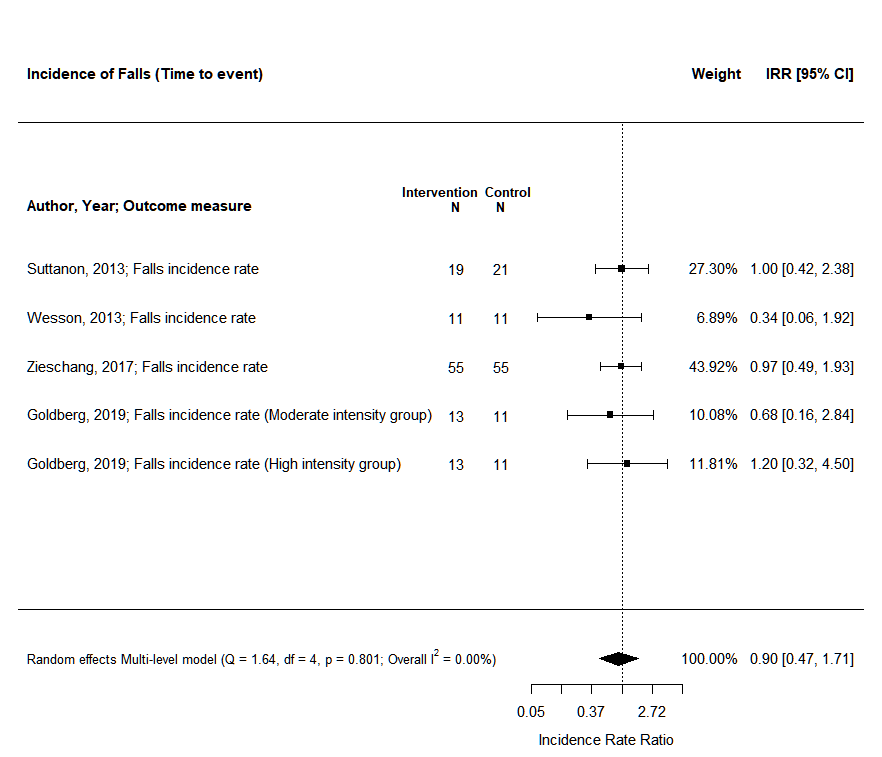
 
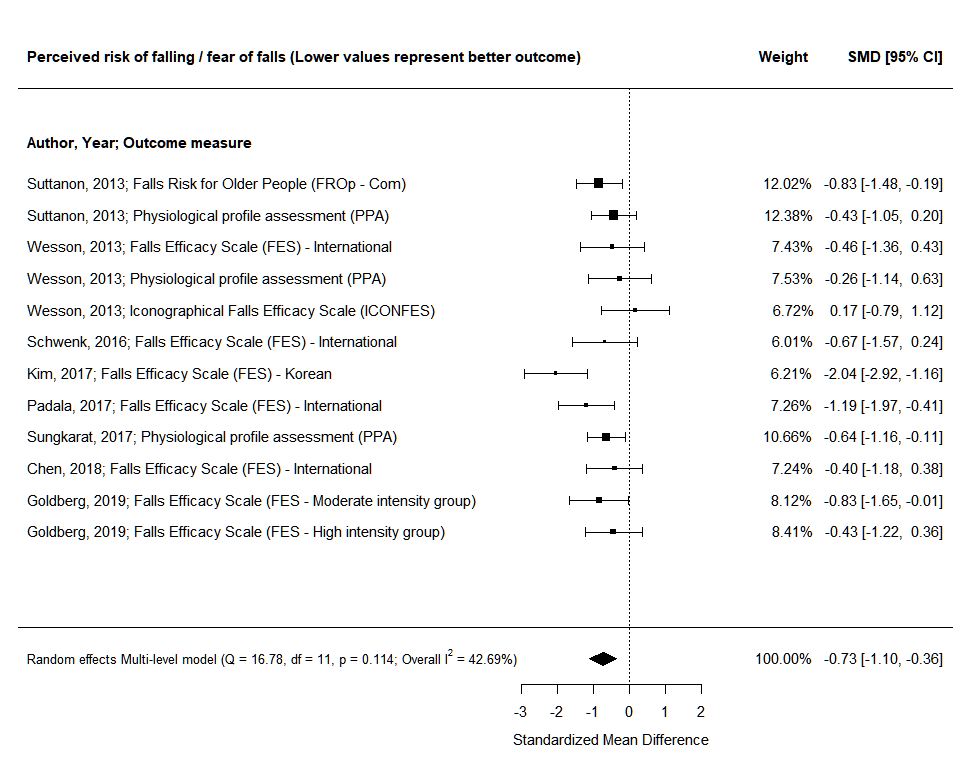


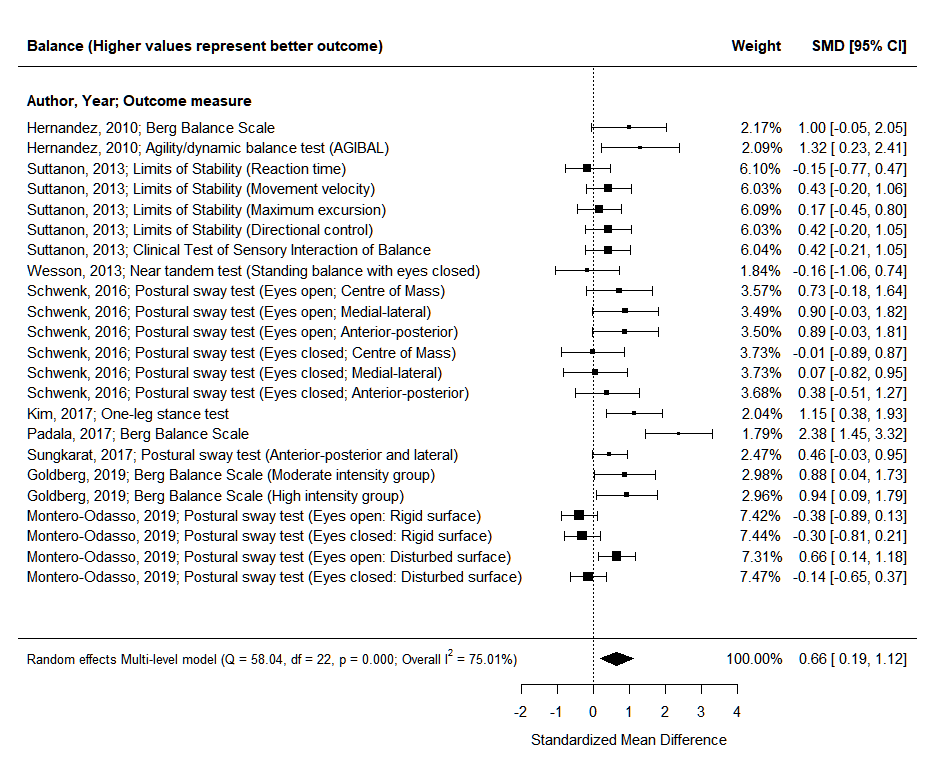


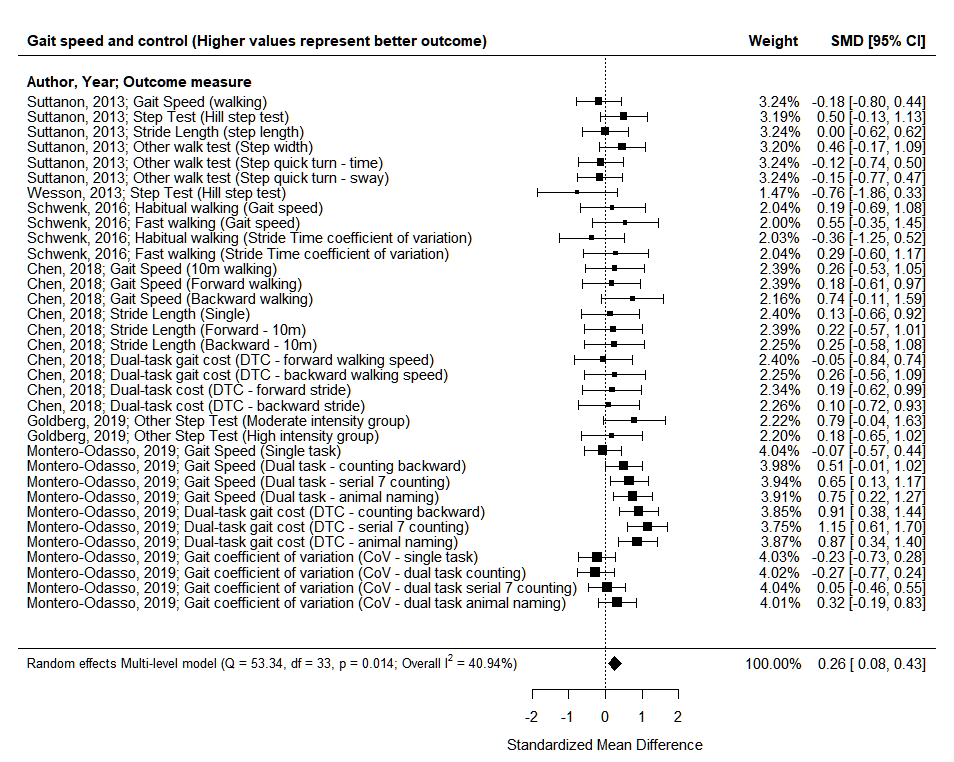


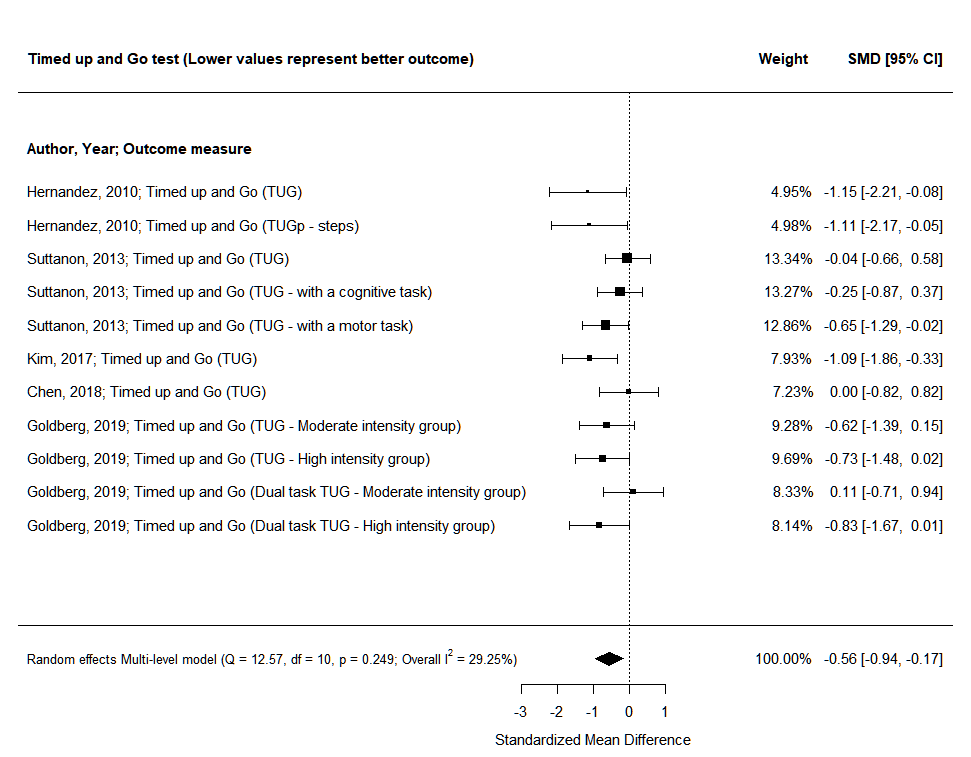


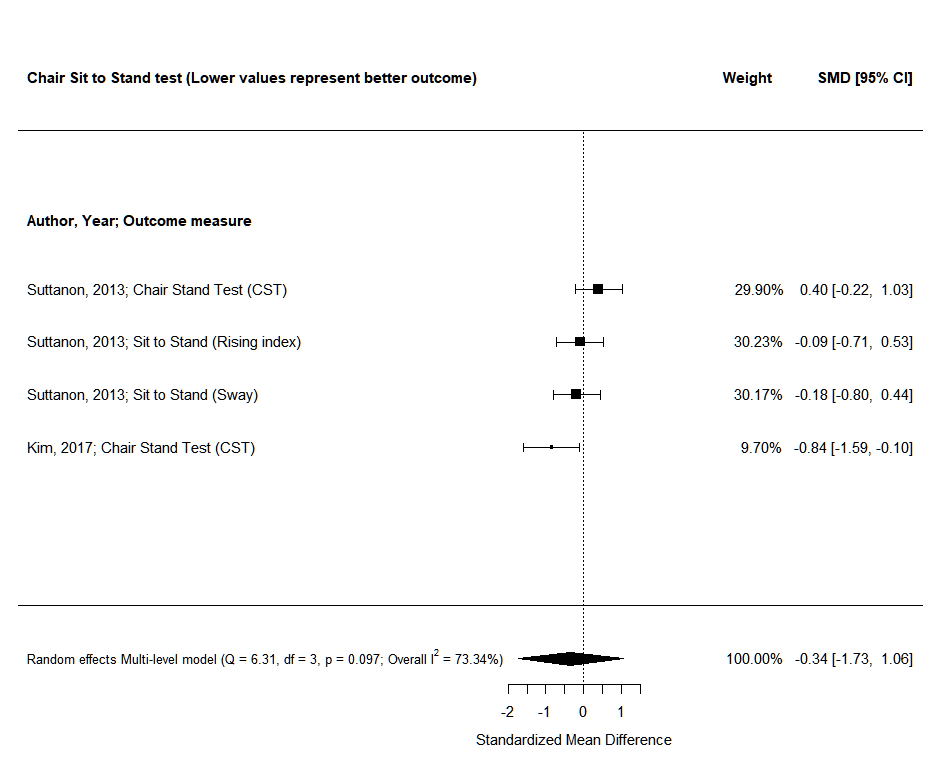

Supplement: Supplementary file 5 — Additional file 5. [file 12877_2021_2641_MOESM5_ESM.docx]
