# Supplementary material for: Fall prevention in community-dwelling adults with mild to moderate cognitive impairment: a systematic review and meta-analysis
Source: BMC Geriatr. 2021 Dec 10;21:689. doi: 10.1186/s12877-021-02641-9 (PMC8665555; doi:10.1186/s12877-021-02641-9)
Supplement: Supplementary file 6 — Additional file 6. [file 12877_2021_2641_MOESM6_ESM.docx]

**Additional File 6: Forest Plots for Subgroup Analysis by Intervention Type**


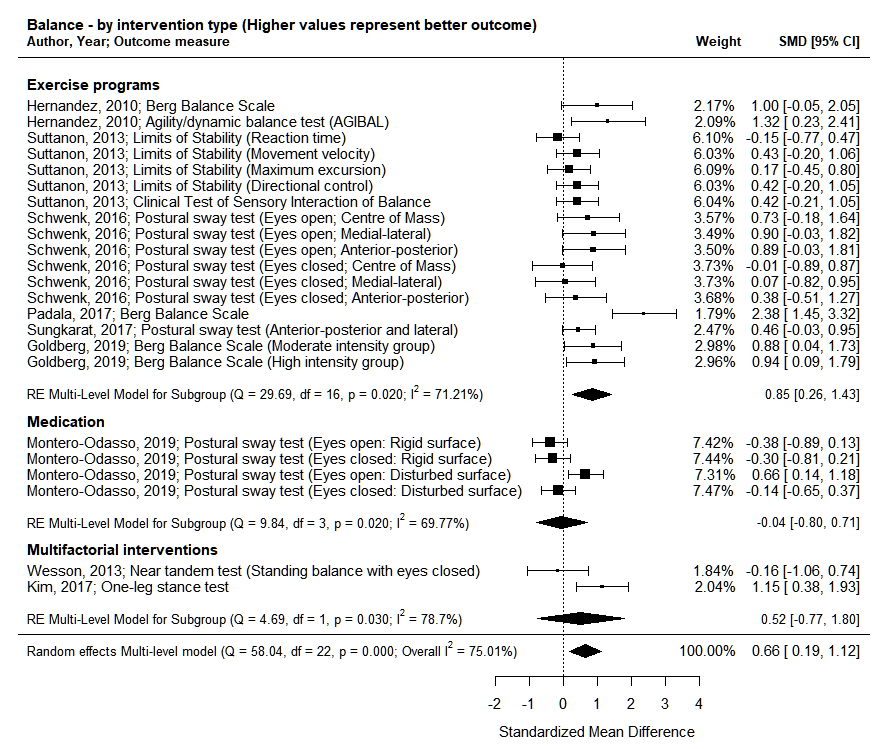


**Test for differences between groups (based on Intervention type): p=0.44 (non-significant)**


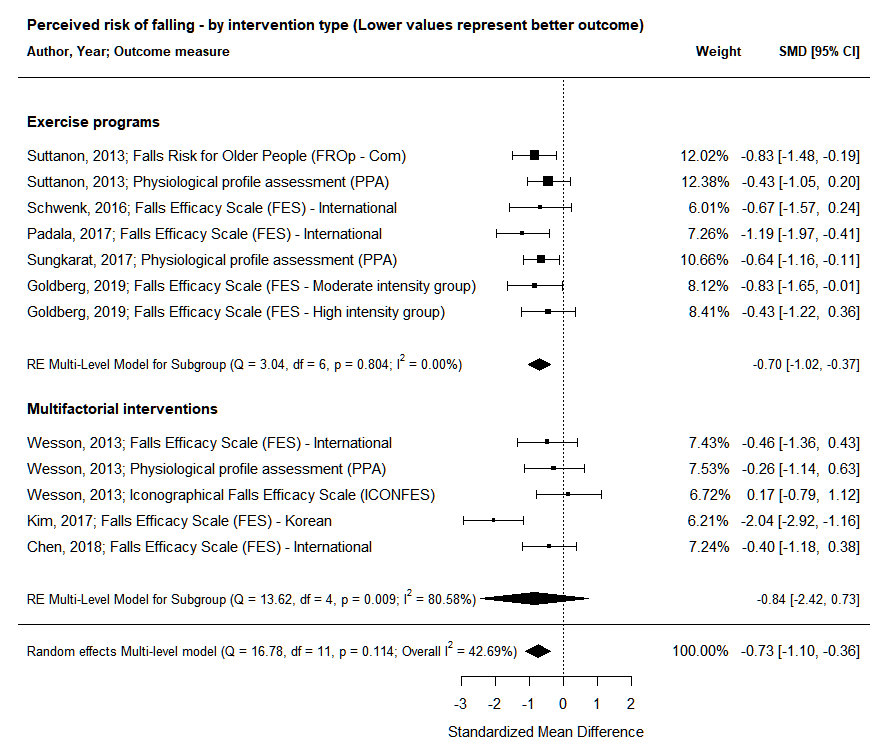


**Test for differences between groups (based on Intervention type): p=0.93 (non-significant)**


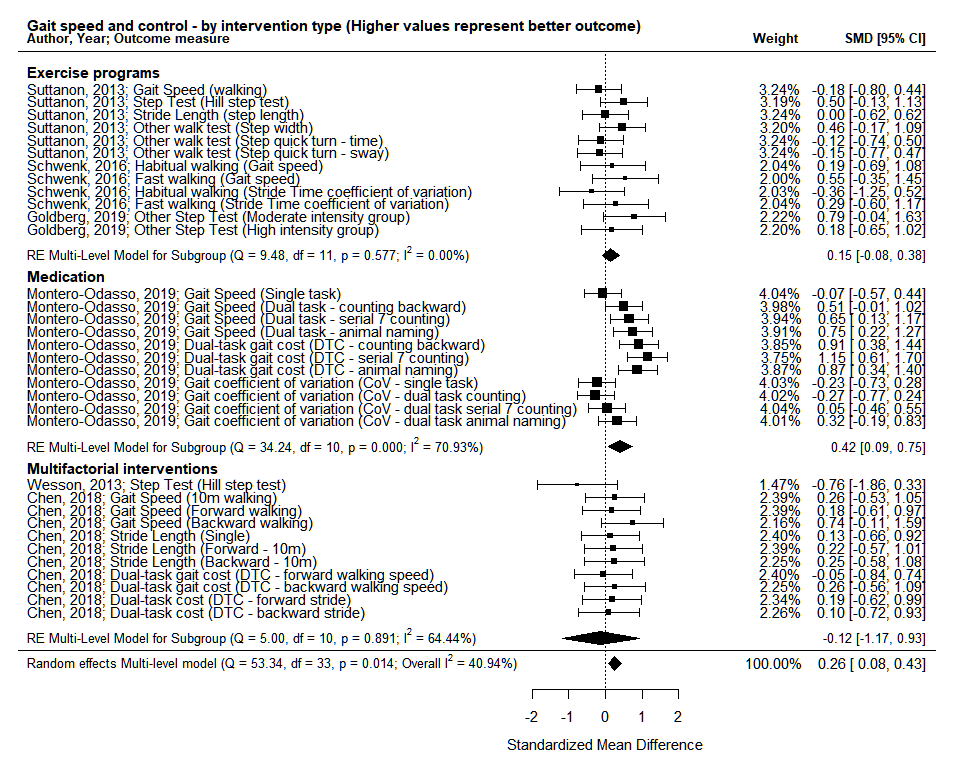


**Test for differences between groups (based on Intervention type): p=0.27 (non-significant)**
